# Proximity to Sports Facilities and Sports Participation for Adolescents in Germany

**DOI:** 10.1371/journal.pone.0093059

**Published:** 2014-03-27

**Authors:** Anne K. Reimers, Matthias Wagner, Seraphim Alvanides, Andreas Steinmayr, Miriam Reiner, Steffen Schmidt, Alexander Woll

**Affiliations:** 1 Department of Sport Science, University of Konstanz, Konstanz, Germany; 2 Institute of Sport and Sport Sciences, Karlsruhe Institute of Technology, Karlsruhe, Germany; 3 Department of Geography and the Built Environment, Northumbria University, Ellison Building, Newcastle-upon-Tyne, United Kingdom; 4 Swiss Institute for Empirical Economic Research (SEW), University of St. Gallen, St. Gallen, Switzerland; University of Sao Paulo, Brazil

## Abstract

**Objectives:**

To assess the relationship between proximity to specific sports facilities and participation in the corresponding sports activities for adolescents in Germany.

**Methods:**

A sample of 1,768 adolescents aged 11–17 years old and living in 161 German communities was examined. Distances to the nearest sports facilities were calculated as an indicator of proximity to sports facilities using Geographic Information Systems (GIS). Participation in specific leisure-time sports activities in sports clubs was assessed using a self-report questionnaire and individual-level socio-demographic variables were derived from a parent questionnaire. Community-level socio-demographics as covariates were selected from the INKAR database, in particular from indicators and maps on land development. Logistic regression analyses were conducted to examine associations between proximity to the nearest sports facilities and participation in the corresponding sports activities.

**Results:**

The logisitic regression analyses showed that girls residing longer distances from the nearest gym were less likely to engage in indoor sports activities; a significant interaction between distances to gyms and level of urbanization was identified. Decomposition of the interaction term showed that for adolescent girls living in rural areas participation in indoor sports activities was positively associated with gym proximity. Proximity to tennis courts and indoor pools was not associated with participation in tennis or water sports, respectively.

**Conclusions:**

Improved proximity to gyms is likely to be more important for female adolescents living in rural areas.

## Introduction

Despite considerable evidence on the health benefits of regular physical activity [1], [2], few adolescents worldwide as well as in Germany engage in sufficient levels of physical activity [3], [4]. Individual-level socio-demographic disparities in physical activity participation have been well-established. Adolescents from families with low socioeconomic status [5]–[8] and non-native ethnic background [9]–[11] appear to be less active than those with medium or high socioeconomic status and native adolescents, respectively.

In addition to individual characteristics, environmental factors are assumed to explain disparities in physical activity participation [12], [13]. On a theoretical basis, ecological models highlight the importance of the environment in relation to physical activity behavior [14]–[16]. These models posit that the neighborhood or community environment exhibits several features such as recreational facilities, aesthetic design and public spaces that hinder or promote the residents' physical activity [15], [17], [18]. Proximity to sports facilities is one important environmental resource that may predict participation in physical activity [19]. Two theoretical processes may determine the relationship between availability or proximity and utilization of sports facilities [20]. First, the absence of nearby sports facilities in the community increases the effort of residents to participate in physical activities that require these facilities or makes it impossible to participate in such activities when one is unable to reach a more distant facility. Thus, limited availability or low proximity to sports facilities may discourage their usage. This is particularly the case for adolescents because they are more independent from their parents than younger children and may be expected to organize their leisure-time by themselves [21]. The second theoretical process postulates that proximity to sports facilities may generate new demand for their use. Adolescents living close to a sports facility may see it on a daily basis and this familiarity could generate demand for individual visits, as well as for partaking in organised sports club activities taking place in these facilities.

In the geographical literature a distinction has been established between the terms availability and proximity [22]. Proximity is related to the concept of accessibility, in other words how easy it is to access a specific destination, in relation to physical distance or cost in general (e.g. financial or time resources). Availability, on the other hand, is referring to the number or density of destinations and has been utilized as a measure of “exposure” to resources in physical activity studies [23]. There are also methodological differences in the way the physical proximity aspects of accessibility can be measured (e.g. by street network or straight line distance), but these are likely to have very little impact for the relatively short distances examined here, as demonstrated by Burgoine and colleagues [24]. In the current study we implement the concept of accessibility to (rather than availability of) potential destinations by calculating and applying a measure of physical proximity to the nearest sports facilities.

Since the relationship between physical activity and the physical environment is assumed to be context- and behavior-specific [25], proximity to specific sports facilities is assumed to influence sport activities taking place in such facilities. For example, the proximity to indoor pools is supposed to influence water sports activities taking place in indoor pools such as leisure-time swimming or playing water polo for a sports club. Some evidence suggests that adolescents having better availability of or proximity to sports facilities are more likely to be physically active than adolescents with poor availability of sports facilities. Results from studies conducted in the U.S. [26], [27], Hong Kong [28], Australia [29] and the Netherlands [30]–[32] showed that availability of sports facilities was associated with physical activity participation in adolescents. For Greenlandic adolescents, availability of indoor sports facilities showed a positive association with vigorous physical activity, but was negatively associated with moderate-to-vigorous physical activity [33]. For children in Germany, while in rural areas distance to the nearest sports facility was negatively associated with sports activities, no relationship was found in urban areas [34].

To date there are no studies of adolescents linking proximity to specific facilities with the corresponding sports activities that normally take place in such facilities. Additionally, the relationship between proximity to sports facilities and sports participation has not been studied in German adolescents. Thus, the aim of this study was to assess the relationship between proximity to specific sports facilities and participation in the corresponding sports activities for adolescents in Germany. It was hypothesized that proximity to specific sports facilities is positively related to participation in the corresponding leisure-time sports activities.

It is important to note here the distinction between a sports club and a sports facility in the German context. Sports clubs (translated from the German term “Vereine”) are associations or unions which offer exercise programs (often for competitive sports), and refer to the administrative aspect of organised sports. Sports facilities are referring to the physical infrastructure (e.g. swimming pools, tennis cours and gyms) where sports clubs offer a range of competitive sports. In Germany there are roughly 90 000 sports clubs, which are particularly important settings for being physically active [35], [36] and a substantial proportion of adolescents' physical activity takes place in organized sports [35]. Thus, the current study focuses on sports activity participation in sports clubs, in relation to proximity to sports facilities.

Additionally, physical activity in sports clubs contributes the highest proportion of vigorous physical activity compared to other leisure activities and school physical education [37] and as a result may have a higher impact on health [1]. Hence, investigating the association between proximity to sports facilities and sports activities in sports clubs is particularly important for our understanding of area-based disparities in adolescents' physical activity in Germany.

## Materials and Methods

### Sampling and participants

The study data set was collated from the Motorik Modul (MoMo Study) [38] and the German Health Interview and Examination Survey for Children and Adolescents (KiGGS). The MoMo Study is a nationwide study on physical fitness and physical activity habits for children and adolescents in Germany and is part of the KiGGS [37], [39]. To ensure a diverse sample of children and adolescents between 4 and 17 years old a nationwide stratified multi-stage probability sample with three evaluation levels was drawn for both studies [39]–[41]. First, a systematic sample of 167 primary sampling units was selected from an inventory of German communities stratified according to the BIK classification system [42] that measures the level of urbanization and the geographic distribution of the population [39]. The probability of any community being picked was proportional to the number of inhabitants younger than 18 years. Second, an age stratified sample of randomly selected children and adolescents was drawn from the official registers of local residents for the KiGGS. Third, a subsample of the KiGGS was randomly assigned to the MoMo Study. The MoMo Study includes complete data sets from 1828 adolescents. The sample used here was further restricted to the participants selected from 161 communities (out of the 167 primary sampling units) for which data sources on sports facilities were available, resulting in a total of 1768 adolescents.

The KiGGS and the MoMo Study were approved by the Charité/Universitätsmedizin Berlin ethics committee and the Federal Office for the Protection of Data and were conducted according to the Declaration of Helsinki [43].

### Data sources

The MoMo baseline data was collected between 2003 and 2006 [38]. Parents and adolescents were invited to the examination rooms located at central locations of the 167 cities and municipalities (primary sampling units) that were within close proximity of their homes. Parents and adolescents gave written informed consent and the adolescents answered the questionnaires in the presence of a qualified interviewer on site [41]. Socio-demographic characteristics were assessed using a parent questionnaire and information on sports activities was assessed using an adolescent self-report questionnaire. Furthermore, objective data on socioeconomic environments was drawn from another database, the INKAR database (Indikatoren und Karten zur Raum- und Stadtentwicklung; indicators and maps on spatial development) which is provided by the Federal Office for Building and Regional Planning. INKAR contains a wide range of regional variables, for instance regarding the composition of population, employment rates, levels of education, production and wages [44]. The INKAR data is available for several geographical levels and for the current study the community level was chosen.

Distances to the nearest sports facilities were objectively measured using a Geographical Information System (GIS) [34]. The address and type of different sports facilities were collected from various sources for 161 out of the 167 primary sampling units of the MoMo-Study. The main source of information was the official municipal websites, where most municipalities provide registers of available facilities. These were complemented with addresses from websites of local sports associations as well as from Google Maps. For communities where the relevant information could not be accessed online, lists of sports facilities were requested from the municipal administration. The home addresses of the survey participants and those of the sports facilities were geocoded using Microsoft MapPoint Europe 2010 in combination with IC-Tools, a MapPoint AddIn for Microsoft Excel. For addresses where MapPoint was not able to determine the exact coordinates, Google Maps and Bing Maps were used instead. Linear distances between home addresses and facilities were calculated using the STATA module globdist.

### Measures

#### Proximity to sports facilities within the community

To determine proximity to sports facilities within the community, for each participant the linear distance from their home address to the nearest sports facility within the community was calculated. Thus, three proximity variables were created reflecting the distances to the nearest gym, tennis court, and indoor pool.

#### Participation in sports activities

Participation in specific activities in sports clubs was measured using the MoMo-PAQ [45]. In this questionnaire sports club membership was measured using one item: “Are you a member of a sports club?” (Responses: (1) Yes, I am currently a member of one sports club. (2) Yes, I am currently a member of several sports clubs. (3) I used to be a member of a sports club, but not anymore. (4) No, I have never been a member of a sports club). This measure was dichotomized and the new variable discriminates between members (combining response options 1 and 2) and non-members (response options 3 and 4). Furthermore, participants were asked to report at most four different sports they engage in at a sports club. Based on these items three new variables were computed that differentiate between non-members of sports clubs and members of sports clubs that (1) engage in sports taking place in gyms (e.g. gymnastics, handball, volleyball etc.); (2) engage in water sports taking place in indoor pools (e.g. swimming, water polo); (3) engage in tennis.

#### Covariates

Individual-level socioeconomic status was calculated separately for both parents combining their responses on educational and professional status and total household income [46]. These three aspects (income, educational and professional status) were scored on a scale from 1 to 7 and a sum score was created (range: 3–21) and categorized into low (3–8), medium (9–14) or high (15–21) socioeconomic status [47]. The higher score for either of the parents was used and adolescents with separated parents were assigned the socioeconomic status of the parent they lived with. Migration background was assumed if the adolescent had immigrated to Germany, or at least one parent was not born in Germany, or if both parents immigrated to Germany or had non-German nationality [39], [48]. Level of urbanization is an area-level variable describing the population size of the community (rural area or small town: ≤19,999 residents; urban area: ≥20,000 residents) and was assigned to the primary sample units according to the BIK classification system [39]. Unemployment rate is also an area-level variable assigned to the primary sample units and calculated as the percentage of unemployed inhabitants of employable age. This variable was selected as an indicator of the community-level socioeconomic environment from the INKAR data set [44].

### Statistical analysis

The associations of distances with specific sports facilities and leisure-time sports activities in sports clubs were analyzed using logistic regression analyses separately for both genders. In order to account for the hierarchical structure of the data with subjects nested within communities (primary sample units) multilevel analysis with community and individual as levels was considered [49]. However, since no significant clustering in communities was found in the unconditional (null) models (boys: z = 1.506, p = 0.132; girls: z = 1.332, p = 0.183), we decided to conduct single-level logistic regression analyses for individuals without adjustments for clustering at the sample point level of communities. We calculated four models, with model 1 disclosing the relationship of the covariates (age, socioeconomic status, migration background, unemployment rate, level of urbanization) with the outcome variable and model 2 disclosing the unadjusted effects of distances to specific sports facilities on the respective sports activities. Model 3 is the full model with both the covariates and the distances as predictors. In model 4 we included the interaction term of “distance * level of urbanization” because Steinmayr and colleagues [34] showed that the strength of the relationship of distances to the nearest sports facility and sports activity differed between rural and urban areas. In order to prevent multi-collinearity in the interaction models, continuous variables were mean centered in the logistic regression analysis. To decompose significant interaction terms the PROCESS macro from Hayes [50] was run.

All analyses were carried out in IBM Statistical Package for Social Science (SPSS) version 20 (IBM, New York, USA)

## Results

### Description of the sample

Descriptive data of individual and community-level correlates and straight line distances to the nearest sports facilities are presented in Table 1. The sample consisted of 865 girls and 903 boys, with a mean age 14.21 (SD = 1.94). Approximately half of the sample had medium socioeconomic status and one out of four had either high or low socioeconomic status, respectively. Nearly 11% of the participants had migration background, while 56.0% resided in rural and 44.0% in urban areas. 47.9% of girls and 59.2% of boys participated in sports club activities. The shortest mean distance was to the nearest gym (1.26 km; SD = 1.53), followed by the mean distance to the nearest tennis court (2.13 km; SD = 2.18). The mean distance to the nearest indoor pool was the longest at 4.12 km (SD = 5.01).

**Table 1:** Sample description.

	girls (n = 865)	boys (n = 903)	total (n = 1768)
*Individual-level correlates n (%)*			
Socioeconomic status			
low	238 (27.9)	220 (24.7)	458 (26.3)
medium	416 (48.8)	453 (50.9)	869 (49.9)
high	198 (23.2)	217 (24.4)	415 (23.8)
Migration background			
no	758 (87.7)	818 (90.6)	1576 (89.2)
yes	106 (12.3)	85 (9.4)	191 (10.8)
Participation in sports club activities			
no	450 (52.1)	367 (40.8)	817 (46.4)
yes	413 (47.9)	532 (59.2)	945 (53.6)
*Community-level correlates*			
Level of urbanization n (%)			
rural	477 (55.1)	513 (56.8)	990 (56.0)
urban	388 (44.9)	390 (43.2)	778 (44.0)
Unemployment rate M±SD	7.3 ±4.0	7.2 ±3.9	7.2 ±3.9
*Straight line distances (in kilometers) to the closest sports facilities M±SD*			
straigt line distance to gym	1.21 ±1.43	1.31 ±1.61	1.26 ±1.53
straight line distance to tennis court	2.17 ±2.12	2.09 ±2.25	2.13 ±2.18
straigt line distance to indoor pool	3.96 ±4.84	4.28 ±5.17	4.12 ±5.01

Note: numbers may not add to the full sample sizes due to missing values.

### Relationship between proximity to specific sports facilities and participation in the corresponding sports activities

**Figure 1:**
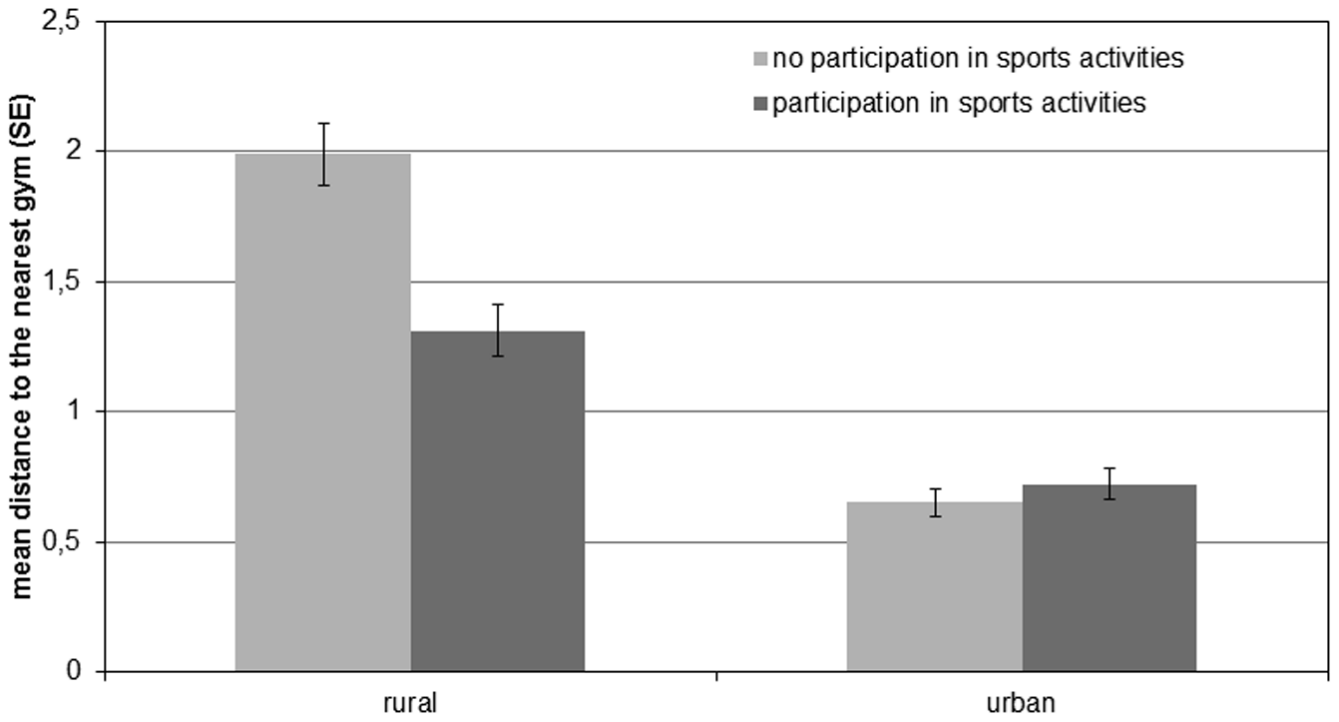
Visual presentation of the interaction of distances to the nearest gym and level of urbanization on leisure-time sports activities taking part in gyms in girls.

Descriptives on participation in specific sports activities are presented in Table 2. The logisitic regression analysis showed that distances (in kilometers) to the nearest tennis court and to the nearest indoor pool were not significantly related to the respective sports activites (tennis playing, swimming or polo) for girls or for boys in any of the Models 2–4 (data not shown here). Concerning the distances to the nearest gym, regression analysis revealed that girls having longer distances were less likely to do sports activities that require access to a gym (Table 3). The distance to the nearest gym was negatively associated with participation in sports activities in the unadjusted regression model (Model 2) and the multiple regression model (Model 3) indicating that an increase of one standard deviation ( = 1.43 km) in distance to the nearest gym reduces the odds of participating in sports activities in sports clubs by 24%. Model 4 revealed that distance to the nearest gym significantly interacted with level of urbanization. The PROCESS macro from Hayes [50] that was run to better interpret this interaction term showed a negative association of distance and participation in sports activities for girls living in rural areas (p<0.001) but no significant relationship for girls living in urban areas (p = 0.393) (Figure 1). For boys, distances to the nearest gym were not associated with leisure-time sports activities that require access to a gym (Table 4).

**Table 2:** Descriptive data on sports participation and distances (in kilometers) to the nearest sports facilities.

	no sports	indoor sports	tennis	water sports
*Girls n (% of female sample)*	466 (53.9)	366 (42.3)	30 (3.5)	51 (5.9)
Straigt line distance to gym M±SD	1.33 ±1.56	1.07 ±1.22	1.42 ±1.82	0.81 ±0.86
Straight line distance to tennis court M±SD	2.33 ±2.22	2.03 ±2.04	1.76 ±2.32	1.56 ±1.51
Straigt line distance to indoor pool M±SD	4.06 ±5.09	3.97 ±4.78	5.81 ±7.18	3.64 ±4.14
*Boys n (% of male sample)*	380 (42.1)	483 (53.5)	42 (4.7)	48 (5.3)
Straigt line distance to gym M±SD	1.33 ±1.62	1.26 ±1.57	1.10 ±0.89	1.27 ±1.45
Straight line distance to tennis court M±SD	2.10 ±2.18	2.09 ±2.32	1.80 ±2.15	1.97 ±1.76
Straigt line distance to indoor pool M±SD	4.44 ±5.26	4.33 ±5.31	4.17 ±4.92	3.28 ±3.06

Note: numbers may not add to the full sample sizes due to missing values.

**Table 3:** Associations of socio-demographic variables and distances to the nearest gym with participation in leisure-time indoor sports for girls (N = 754).

	Model 1 OR (95%CI)	Model 2 OR (95%CI)	Model 3 OR (95%CI)	Model 4 OR (95%CI)
unemployment rate	.91 (.87; .94)		.91 (.87; .95)	.91 (.87; .95)
rural^a^	1.04 (.75; 1.43)		1.25 (.89; 1.76)	1.07 (.74; 1.54)
low SES^b^	.43 (.27; .66)		.44 (.29; .69)	.45 (.29; .70)
medium SES^b^	.87 (.60; 1.27)		.91 (.62; 1.33)	.91 (.62; 1.33)
no migration background^c^	1.84 (1.11; 3.02)		1.95 (1.18; 3.23)	2.03 (1.23; 3.37)
age	.90 (.83; .97)		.89 (.83; .97)	.89 (.82;.96)
distance to gym^d^		.82 (.70; .96)	.76 (.64; .91)	1.22 (.71; 1.93)
distance to gym^d^ * level of urbanization				.58 (.35; .95)
Nagelkerkes Pseudo-R^2^	.104	.012	.120	.128

Note: ^a^ reference =  urban; ^b^ reference =  high; ^c^ reference =  migration background; ^d^ means centered; OR = Odds ratio, CI = confidence interval; bold values represent statistically significant associations (p≤0.05); italic values show significant interaction terms (p≤0.10).

**Table 4:** Associations of socio-demographic variables and distances to the nearest gym with participation in leisure-time indoor sports for boys (N = 815).

	Model 1 OR (95%CI)	Model 2 OR (95%CI)	Model 3 OR (95%CI)	Model 4 OR (95%CI)
unemployment rate	.90 (.86;.93)		.90 (.86; .93)	.90 (.86; .93)
rural^a^	1.06 (.78; 1.45)		1.08 (.77; 1.50)	1.14 (.80; 1.61)
low SES^b^	.41 (.26; .63)		.41 (.26; .63)	.41 (.26; .63)
medium SES^b^	.65 (.45; .94)		.65 (.45; .95)	.65 (.45; .95)
no migration background^c^	1.49 (.88; 2.52)		1.50 (.89; 2.54)	1.52 (.90; 2.58)
age	.88 (.81;.95)		.88 (.81;.95)	.88 (.85;.95)
distance to gym^d^		.96 (.83; 1.10)	.98 (.84; 1.15)	.81 (.52; 1.26)
distance to gym^d^ * level of urbanization				1.25 (.78; 1.20)
Nagelkerkes Pseudo-R2	.108	.001	.108	.109

Note: ^a^ reference =  urban; ^b^ reference =  high; ^c^ reference =  migration background; ^d^ means centered; OR = Odds ratio, CI = confidence interval; bold values represent statistically significant associations (p≤0.05).

## Discussion

This nationwide study provides empirical evidence that girls from rural areas with better gym availability were more likely to participate in leisure-time indoor sports activities (e.g. dancing, volleyball, gymnastics, etc.).

Concerning the relationship between proximity to sports facilities and participation in sports clubs, only for adolescent girls from rural areas was gym proximity positively related to participation in indoor sports activities. The proximity to gyms was not associated with indoor sports activities for adolescent boys and no association was found between proximity to tennis courts and indoor pools and respective sports activities for either gender. Previous studies that do not distinguish the different types of sports facilities have been conflicting. While two studies conducted in Australia [29] and in the Netherlands [31] found no association between availability of sports facilities and sports participation, other studies from Europe [30], [33], the U.S. [26], [27] and Hong Kong [28] revealed positive relationships. The conflicting findings are likely to occur because availability of certain types of facilities may have an impact on sports participation while the availability of others may have no impact, as shown in the current study. Furthermore, no study has associated proximity to specific sports facilities with respective sports activities that normally take place in these facilities. Adolescents having low proximity to a specific type of facility might have compensated by engaging in other sports activities for which they can use other facilities available to them. Thus, overall sports participation may not be associated with proximity to a specific type of facility. In the current study proximity to indoor pools and tennis courts was not associated with the respective sports activities (e.g. tennis, swimming, water polo) which are in any case less common in Germany (<10%). Thus, adolescents choosing to participate in these activities may have strong preferences leading to this decision (e.g. for water sports), which could result in engaging with these sports irrespective of constraints in reaching the respective sports facilities.

The association between gym availability and indoor sports participation was only significant for girls from rural areas, but not for boys or for girls from urban areas. Powell and colleagues [26] also showed in a study conducted in the U.S. that only for female adolescents was proximity to commercial physical activity-related facilities associated to physical activity. Low proximity to sports facilities could be a barrier of participation in sports activities, especially in adolescent girls, because they tend to depend on others for their daily mobility and therefore experience more difficulty in reaching sports facilities further away from their home. Distinguishing between rural and urban areas may be appropriate in samples from countries with distinct rural-urban differences like Germany. To the best of our knowledge urban-rural differences have not been assessed in other studies, in the context of this research, but some studies were only based on participants from larger cities [28]–[30]. Although in Hong Kong [28] and Rotterdam/Netherlands [30] positive relationships were observed for availability of sports activities and physical activity, a stronger association may occur in rural areas. Since in rural areas public transport and infrastructure tend to be poorer, available facilities can be less accessible and sports participation may depend more on proximity to sports facilities. Furthermore, because in urban areas there is a higher density of sports facilities such as gyms, tennis courts and indoor pools, adolescents living there may have adequate proximity to sports facilities in general; thus, no association of proximity to sports facilities and sports participation can be observed in urban areas. Nevertheless, parents of middle school youth living in both urban and rural areas have commented that distance to sports facilities is one of the primary barriers to physical activity [51].

### Strengths and limitations

The strengths of the current study are that it is based on objective measures of distances to the nearest sports facilities and on a nationwide diverse sample of adolescents encompassing a broad age range. Additionally, in this study we collected data on different types of sports facilities and were able to associate the proximity to specific sports facilities with respective sports activities usually taking place in such facilities. However, the results of this study should be interpreted with caution because of some limitations. First, this is a cross-sectional study that does not allow for causal inferences of relationships. Second, participation in specific leisure-time sports activities has been assessed using a self-report questionnaire which might question the validity of the measure. Third, we did not collect information matching the specific facilities used by each sports club that provides sports programs for adolescents. Thus, we cannot assume that sports club activity programs for adolescents were offered at the nearest sports facility. Furthermore, we cannot be certain that the adolescents participating in specific leisure-time sports activities use the nearest sports facility or another facility. Finally, we did not assess other attributes of the sports facilities, such as size or attractiveness that may also influence sports participation.

## Conclusions

The results of the current study show that improved gym availability is likely to be more important for female adolescents living in rural areas. Although this study did not aim to fully explain community variance for adolescents' participation in specific leisure-time sports activities in sports clubs, there was unexplained community-level variance in the regression models. Additional community-level factors could be relevant in explaining sports participation, such as geographical distance and monetary or time costs. We suggest that in order to understand community-level influences on sports participation, the impact of, for example, accessibility to sports facilities should be investigated in further studies.

## References

[1] Janssen I, Leblanc AG (2010) Systematic review of the health benefits of physical activity and fitness in school-aged children and youth. International Journal of Behavioral Nutrition and Physical Activity 7: 40. 20459784 10.1186/1479-5868-7-40 PMC2885312

[2] Biddle SJ, Asare M (2011) Physical activity and mental health in children and adolescents: a review of reviews. Br J Sports Med 45: 886–895. 21807669 10.1136/bjsports-2011-090185

[3] Hallal PC, Andersen LB, Bull FC, Guthold R, Haskell W, et al (2012) Global physical activity levels: surveillance progress, pitfalls, and prospects. Lancet 380: 247–257. 22818937 10.1016/S0140-6736(12)60646-1

[4] Jekauc D, Reimers A, Wagner MO, Woll A (2012) Prevalence and socio-demographic correlates of the compliance with the physical activity guidelines in children and adolescents in Germany. BMC Public Health 12: 714. 22935245 10.1186/1471-2458-12-714 PMC3489607

[5] Brodersen NH, Steptoe A, Boniface DR, Wardle J (2007) Trends in physical activity and sedentary behaviour in adolescence: ethnic and socioeconomic differences. British Journal of Sports Medicine 41: 140–144. 17178773 10.1136/bjsm.2006.031138 PMC2465219

[6] Hanson MD, Chen E (2007) Socioeconomic status and health behaviors in adolescence: A review of the literature. Journal of Behavioral Medicine 30: 263–285. 17514418 10.1007/s10865-007-9098-3

[7] Yang X, Telama R, Laakso L (1996) Parents' physical activity, socioeconomic status and education as predictors of physical activity and sport among children and youth – A 12-year follow-up study. International Review for the Sociology of Sport 31: 273–294.

[8] Telama R, Laakso L, Nupponen H, Rimpela A, Pere L (2009) Secular Trends in Youth Physical Activity and Parents' Socioeconomic Status From 1977 to 2005. Pediatric Exercise Science 21: 462–474. 20128365 10.1123/pes.21.4.462

[9] Brug J, van Stralen MM, Chinapaw MJ, De Bourdeaudhuij I, Lien N, et al (2012) Differences in weight status and energy-balance related behaviours according to ethnic background among adolescents in seven countries in Europe: the ENERGY-project. Pediatr Obes 7: 399–411. 22730265 10.1111/j.2047-6310.2012.00067.x

[10] Owen CG, Nightingale CM, Rudnicka AR, Cook DG, Ekelund U, et al (2009) Ethnic and gender differences in physical activity levels among 910-year-old children of white European, South Asian and AfricanCaribbean origin: the Child Heart Health Study in England (CHASE Study). International Journal of Epidemiology 38: 1082–1093. 19377098 10.1093/ije/dyp176 PMC2720395

[11] Sagatun A, Kolle E, Anderssen SA, Thoresen M, Sogaard AJ (2008) Three-year follow-up of physical activity in Norwegian youth from two ethnic groups: associations with socio-demographic factors. Bmc Public Health 8: 419. 19102770 10.1186/1471-2458-8-419 PMC2640384

[12] Ball K, Timperio AF, Crawford DA (2006) Understanding environmental influences on nutrition and physical activity behaviors: where should we look and what should we count? Int J Behav Nutr Phys Act 3: 33. 16999874 10.1186/1479-5868-3-33 PMC1592115

[13] Macintyre S (2007) Deprivation amplification revisited; or, is it always true that poorer places have poorer access to resources for healthy diets and physical activity? International Journal of Behavioral Nutrition and Physical Activity 4: 32. 17683624 10.1186/1479-5868-4-32 PMC1976614

[14] Sallis JF, Owen N, Fisher EB (2008) Ecological models of health behavior. In: Glanz K, Rimer BK, Viswanath K, editors. Health behavior and health education: theory, research, and practice. 4th ed. San Francisco, Calif.: Jossey-Bass.

[15] Swinburn B, Egger G, Raza F (1999) Dissecting obesogenic environments: The development and application of a framework for identifying and prioritizing environmental interventions for obesity. Preventive Medicine 29: 563–570. 10600438 10.1006/pmed.1999.0585

[16] Spence JC, Lee RE (2003) Toward a comprehensive model of physical activity. Psychology of Sport and Exercise 4: 7–24.

[17] Owen N, Leslie E, Salmon J, Fotheringham MJ (2000) Environmental determinants of physical activity and sedentary behavior. Exerc Sport Sci Rev 28: 153–158. 11064848

[18] Pikora T, Giles-Corti B, Bull F, Jamrozik K, Donovan R (2003) Developing a framework for assessment of the environmental determinants of walking and cycling. Soc Sci Med 56: 1693–1703. 12639586 10.1016/s0277-9536(02)00163-6

[19] Grow HM, Saelens BE, Kerr J, Durant NH, Norman GJ, et al (2008) Where Are Youth Active? Roles of Proximity, Active Transport, and Built Environment. Medicine and Science in Sports and Exercise 40: 2071–2079. 18981942 10.1249/MSS.0b013e3181817baa

[20] Jones A, Panter J (2010) Availability and accessibility in physical activity environments. In: Lake AA, Townshend TG, Alvanides S, editors. Obesogenic environments: complexities, perceptions and objective measures. Chichester, West Sussex: Wiley-Blackwell. pp. 41–61.

[21] Davison KK, Jago R (2009) Change in Parent and Peer Support across Ages 9 to 15 yr and Adolescent Girls' Physical Activity. Medicine and Science in Sports and Exercise 41: 1816–1825. 19657287 10.1249/MSS.0b013e3181a278e2 PMC5489408

[22] Thornton LE, Pearce JR, Kavanagh AM (2011) Using Geographic Information Systems (GIS) to assess the role of the built environment in influencing obesity: a glossary. International Journal of Behavioral Nutrition and Physical Activity 8: 71. 21722367 10.1186/1479-5868-8-71 PMC3141619

[23] Roux AVD, Evenson KR, McGinn AP, Brown DG, Moore L, et al (2007) Availability of recreational resources and physical activity in adults. American Journal of Public Health 97: 493–499. 17267710 10.2105/AJPH.2006.087734 PMC1805019

[24] Burgoine T, Alvanides S, Lake AA (2013) Creating 'obesogenic realities'; do our methodological choices make a difference when measuring the food environment? Int J Health Geogr 12: 33. 23816238 10.1186/1476-072X-12-33 PMC3723649

[25] Giles-Corti B, Timperio A, Bull F, Pikora T (2005) Understanding physical activity environmental correlates: increased specificity for ecological models. Exerc Sport Sci Rev 33: 175–181. 16239834 10.1097/00003677-200510000-00005

[26] Powell LM, Chaloupka FJ, Slater SJ, Johnston LD, O'Malley PM (2007) The availability of local-area commercial physical actlivity-related facilities and physical activity among adolescents. American Journal of Preventive Medicine 33: S292–S300. 17884577 10.1016/j.amepre.2007.07.002

[27] Gordon-Larsen P, Nelson MC, Page P, Popkin BM (2006) Inequality in the built environment underlies key health disparities in physical activity and obesity. Pediatrics 117: 417–424. 16452361 10.1542/peds.2005-0058

[28] Wong BYM, Cerin E, Ho SY, Mak KK, Lo WS, et al (2010) Adolescents' physical activity: Competition between perceived neighborhood sport facilities and home media resources. International Journal of Pediatric Obesity 5: 169–176. 19657859 10.3109/17477160903159432

[29] Prins RG, Ball K, Timperio A, Salmon J, Oenema A, et al (2011) Associations between availability of facilities within three different neighbourhood buffer sizes and objectively assessed physical activity in adolescents. Health & Place 17: 1228–1234. 21889390 10.1016/j.healthplace.2011.07.012

[30] Prins RG, Oenema A, van der Horst K, Brug J (2009) Objective and perceived availability of physical activity opportunities: differences in associations with physical activity behavior among urban adolescents. International Journal of Behavioral Nutrition and Physical Activity 6: 70. 19832969 10.1186/1479-5868-6-70 PMC2770555

[31] Prins RG, Mohnen SM, van Lenthe FJ, Brug J, Oenema A (2012) Are neighbourhood social capital and availability of sports facilities related to sports participation among Dutch adolescents? International Journal of Behavioral Nutrition and Physical Activity 9: 99. 22849512 10.1186/1479-5868-9-90 PMC3479015

[32] Prins RG, van Empelen P, Velde SJT, Timperio A, van Lenthe FJ, et al (2010) Availability of sports facilities as moderator of the intention-sports participation relationship among adolescents. Health Education Research 25: 489–497. 20382675 10.1093/her/cyq024

[33] Niclasen B, Petzold M, Schnohr CW (2012) The association between high recreational physical activity and physical activity as a part of daily living in adolescents and availability of local indoor sports facilities and sports clubs. Scandinavian Journal of Public Health 40: 614–620. 23042458 10.1177/1403494812459815

[34] Steinmayr A, Felfe C, Lechner M (2011) The closer the sportier? Children's sports activity and their distance to sports facilities. European Review of Aging and Physical Activity 8: 67–82.

[35] Jekauc D, Reimers AK, Wagner MO, Woll A (2013) Physical activity in sports clubs of children and adolescents in Germany: Results from a nationwide representative survey. Journal of Public Health DOI10.1007/s10389-013-0579-2.

[36] Brettschneider WD (2001) Effects of sport club activities on adolescent development in Germany. European Journal of Sport Science 1.

[37] Woll A, Kurth BM, Opper E, Worth A, Bös K (2011) The 'Motorik-Modul' (MoMo): physical fitness and physical activity in German children and adolescents. Eur J Pediatr 170: 1129–1142. 21318230 10.1007/s00431-010-1391-4

[38] Wagner MO, Bös K, Jekauc D, Karger C, Mewes N, et al. (2014) Cohort Profile: The Motorik-Modul (MoMo) Longitudinal Study - Physical Fitness and Physical Activity as Determinants of Health Development in German Children and Adolescents. International Journal of Epidemiology. In press. 10.1093/ije/dyt098 23847291

[39] Kurth BM, Kamtsiuris P, Hölling H, Schlaud M, Dolle R, et al (2008) The challenge of comprehensively mapping children's health in a nation-wide health survey: design of the German KiGGS-Study. BMC Public Health 8: 196. 18533019 10.1186/1471-2458-8-196 PMC2442072

[40] Kamtsiuris P, Lange M, Schaffrath Rosario A (2007) [The German Health Interview and Examination Survey for Children and Adolescents (KiGGS): sample design, response and nonresponse analysis]. Bundesgesundheitsblatt - Gesundheitsforschung - Gesundheitsschutz 50: 547–556. 17514438 10.1007/s00103-007-0215-9

[41] Hölling H, Kamtsiuris P, Lange M, Thierfelder W, Thamm M, et al (2007) [The German Health Interview and Examination Survey for Children and Adolescents (KiGGS): Study management and conduct of fieldwork]. Bundesgesundheitsblatt - Gesundheitsforschung - Gesundheitsschutz 50: 557–566. 17514439 10.1007/s00103-007-0216-8

[42] BIK Aschpurwis und Behrens GmbH (2001) BIK Regionen: Ballungsräume, Stadtregionen, Mittel-/Unterzentrengebiete. Methodenbeschreibung zur Aktualisierung 2000. Hamburg.

[43] Kurth BM (2007) [The German Health Interview and Examination Survey for Children and Adolescents (KiGGS): an overview of its planning, implementation and results taking into account aspects of quality management]. Bundesgesundheitsblatt - Gesundheitsforschung - Gesundheitsschutz 50: 533–546. 17514437 10.1007/s00103-007-0214-x

[44] Federal Office for Building and Regional Planning (2012) Informationen aus der Forschung des BBSR. Bonn: BBSR.

[45] Jekauc D, Wagner MO, Kahlert D, Woll A (2013) [Reliability and Validity of MoMo-Physical-Activity-Questionnaire for Adolescents (MoMo-AFB)]. Diagnostica 59: 100–111.

[46] Lampert T, Schenk L, Stolzenberg H (2002) [Conceptualization and operationalization of social inequality in The Child and Adolescent Health Survey]. Gesundheitswesen 64 Suppl 1S48–52. 12870216 10.1055/s-2002-39005

[47] Winkler J, Stolzenberg H (2009) [Adjustment of the Social Class Index for application in the German Health Interview and Examination Survey for Children and Adolescents (KiGGS)]. Wismar: HWS-Hochschule Wismar.

[48] Schenk L, Ellert U, Neuhauser H (2007) [Children and adolescents in Germany with a migration background. Methodical aspects in the German Health Interview and Examination Survey for Children and Adolescents (KiGGS)]. Bundesgesundheitsblatt - Gesundheitsforschung - Gesundheitsschutz 50: 590–599. 17514443 10.1007/s00103-007-0220-z

[49] Goldstein H (1995) Multilevel statistical models. London: Edward Arnold.

[50] Hayes AF (2012) PROCESS: A versatile computational tool for observed variable mediation, moderation, and conditional modeling.

[51] Moore JB, Jilcott SB, Shores KA, Evenson KR, Brownson RC, et al (2010) A qualitative examination of perceived barriers and facilitators of physical activity for urban and rural youth. Health Education Research 25: 355–367. 20167607 10.1093/her/cyq004 PMC10170971

